# Meat-Inspector. Examinations to Be Held June 18, 1901

**Published:** 1901-06

**Authors:** 


### COMMUNICATION.

### MEAT-INSPECTOR. EXAMINATIONS TO BE HELD JUNE 18, 1901.

THE United States Civil Service Commission announces that on June 18, 1901, an examination will be held for the position of meat-inspector in any city in the United States where free postal delivery has been established.

During the past three years, in addition to the regular semi-annual a number of special examinations have been held for this position, but the Commission has failed to secure a sufficient number of eligibles to meet the needs of the service, every person who has passed the examination having been offered an appointment. This examination offers a most excellent opportunity to secure employment in the Government service at a salary of from $1200 to $1400 per annum.

The examination which was held on April 23, 1901, did not result in a sufficient number of eligibles to meet the present needs of the service.

Applicants must be graduates of veterinary colleges. Those graduating prior to or during 1897 will be accepted if from colleges having a course of not less than two years in veterinary science, while applicants graduating since that time must be from colleges having a course of not less than three years. These facts must be shown in the application.

Applications received from persons who are not such graduates will be disapproved.

The examination will consist of the subjects mentioned below, which will be weighted as follows :

| Subject. | Weight. |
|---|---|
| 1. Spelling (second grade) | 5 |
| 2. Arithmetic (second grade) | 5 |
| 3. Letter-writing (second grade) | 5 |
| 4. Penmanship | 5 |
| 5. Copying from plain copy (second grade) | 5 |
| 6. Veterinary anatomy and physiology. | 10 |
| 7. Veterinary pathology | 25 |
| 8. Meat-inspection. | 40 |
| Total | 100 |

Information concerning the scope of the examination may be found in Sections 36 and 60 of the *Manual of Examinations*, revised to January 1, 1901. Age-limit, twenty years or over.

From the eligibles resulting from this examination it is expected that certification will at once be made for the purpose of filling a number of existing vacancies in the position of meat-inspector in the service of the Bureau of Animal Industry, Department of Agriculture, at different places throughout the United States, and to other similar vacancies as they may occur.

This examination is open to all citizens of the United States who comply with the requirements and desire to enter the service. All such persons are invited to apply, and applicants will be examined, graded, and certified with entire impartiality, and wholly without regard to any consideration save their ability as shown by the grade attained in the examination. Preference in certification may be given to eligibles who are legal residents of the place or vicinity where the vacancy exists.

Persons who desire to compete should at once apply to the United States Civil Service Commission, Washington, D. C., for application forms 304 and 375, which should be properly executed and promptly forwarded to the Commission. All persons who have been examined for this position during the past year and failed to pass will be allowed re-examination upon filing a new application. All persons who are unable to file their application prior to the date of the examination will be examined, provided their request is received at this office in sufficient time to ship papers to the place of examination selected by them, conditional on the subsequent filing of their application in proper form showing them to be eligible.

